# Visible Light-Cured Glycol Chitosan Hydrogel Containing a Beta-Cyclodextrin-Curcumin Inclusion Complex Improves Wound Healing In Vivo

**DOI:** 10.3390/molecules22091513

**Published:** 2017-09-10

**Authors:** Sun-Jung Yoon, Hoon Hyun, Deok-Won Lee, Dae Hyeok Yang

**Affiliations:** 1 Department of Orthopedic Surgery, Research Institute of Clinical Medicine of Chonbuk National University-Biomedical Research Institute of Chonbuk National University Hospital, Jeonju 54907, Korea; sjyoon_kos@naver.com; 2 Department of Biomedical Sciences, Chonnam National University Medical School, Gwangju 61469, Korea; hhyun@chonnam.ac.kr; 3 Department of Oral and Maxillofacial Surgery, Kyung Hee University Dental Hospital at Gangdong, Kyung Hee University, Seoul 05278, Korea; verycutebear@hanmail.net; 4 Institute of Cell and Tissue Engineering, College of Medicine, The Catholic University of Korea, Seoul 06591, Korea

**Keywords:** wet dressing, glycol chitosan, beta-cyclodextrin, curcumin, inclusion complex, visible light irradiation, open fracture, soft tissue defect, wound

## Abstract

Scarless wound healing is ideal for patients suffering from soft tissue defects. In this study, we prepared a novel wet dressing (β-CD-ic-CUR/GC) based on the visible light-cured glycol chitosan (GC) hydrogel and inclusion complex between beta-cyclodextrin (β-CD) and curcumin (CUR). We also evaluated its efficacy in the acceleration of wound healing as compared to that of CUR-loaded GC (CUR/GC). The conjugation of glycidyl methacrylate (GM) to GC for photo-curing was confirmed by ^1^H-NMR measurement, and the photo-cured GC hydrogel was characterized by the analyses of rheology, swelling ratio, SEM and degradation rate. After visible light irradiation, the surface/cross-sectional morphologies and storage (G′)/loss (G′′) moduli revealed the formation of hydrogel with interconnected porosity. The dressing β-CD-ic-CUR/GC exhibited a controlled release of 90% CUR in a sustained manner for 30 days. On the other hand, CUR/GC showed CUR release of 16%. β-CD acted as an excipient in improving the water-solubility of CUR and affected the release behavior of CUR. The in vivo animal tests including measurement of the remaining unhealed wound area and histological analyses showed that β-CD-ic-CUR/GC may have potential as a wet dressing agent to enhance soft tissue recovery in open fractures.

## 1. Introduction

Wound dressings have been extensively developed for reducing pain, protecting the wound and surrounding tissue, and promoting the moist wound environment that exists in various wounds cause by excision, burns, trauma or orthopedic surgery [1]. Dry gauze dressing is still the most commonly used primary dressing substance, but it has some drawbacks such as the maintenance of moist conditions and weak exudate absorption [2,3]. In addition, a dry dressing can cause pain when it is removed from the wound because it sticks to the wound owing to exudate evaporation, especially soft tissue defects of orthopedic trauma [2,3]. Therefore, numerous studies have focused on the development of wet dressings for overcoming the drawbacks of dry dressings and enhancing the repair of soft tissue defects [2,3].

Amongst the wet wound dressings, the hydrogel form is designed to hold moisture at the wound surface, providing an ideal environment for wound cleansing and autolytic debridement, by the surgical exposure of the whole pathological injury zone and the removal of all necrotic, contaminated or damaged tissue [4]. Photo-polymerization of polymers using a visible light irradiation system is an attractive method for preparing hydrogels because it can easily control temporal and spatial reaction kinetics and rapidly induce a reaction with a single step under mild conditions [5]. In addition, this system is safer than the use of ultra-violet (UV) light, because overexposure to UV light may cause sunburn, and malignant melanoma by DNA damage owing to the formation of free radicals and oxidative stress, and further damage of soft tissue associated with open fractures [6]. Therefore, natural/synthetic polymer hydrogels photo-cured with visible light irradiation have been introduced to prepare tissue-engineered scaffolds for wound healing [7,8,9].

A natural chitosan polymer is a potential material for preparing hydrogel wound dressings because it can control macrophage polarization, which deeply affects wound healing. Despite the merit of chitosan, it has limited clinical use because of its low bioavailability due to poor water solubility [10]. Glycol chitosan (GC) is a water-soluble chitosan derivative and is known to have an antibacterial effect because of its amine group. Thus, GC-based hydrogel can be used as a scaffold for wound healing [8]. Nevertheless, Chun’s group has recently begun to study the efficacy of GC-based hydrogel on wound healing acceleration [8]. The incorporation of drugs or growth factors in hydrogels has been introduced as potential wound dressings for clinical use [8,11]. Compared with synthetic drugs or growth factors, as a natural extract, curcumin (CUR) has no toxicity and has a longer half-life, and it is known to accelerate wound healing owing to its antioxidant, anti-inflammatory, and detoxification function [12]. Despite its merits, the clinical applications of CUR are limited because of its low bioavailability resulting from its poor water-solubility [13]. Beta cyclodextrin (β-CD) can be used as a tool for improving the water-solubility of CUR due to its unique structure [13].

In the present study, we prepared a wet wound dressing based on a visible light-cured GC hydrogel containing the inclusion complex formed between β-CD and CUR, and evaluated its wound healing acceleration effect, compared with that of GC and CUR-loaded GC hydrogels. The in vivo wound healing test was carried out using a soft tissue defect mouse model, and the efficacy was confirmed by measuring the remaining wound area and histological observation of the hydrogel-treated wound defects.

## 2. Results

### 2.1. Proton Nuclear Magnetic Resonance (^1^H-NMR) Analysis

**Figure 1:**
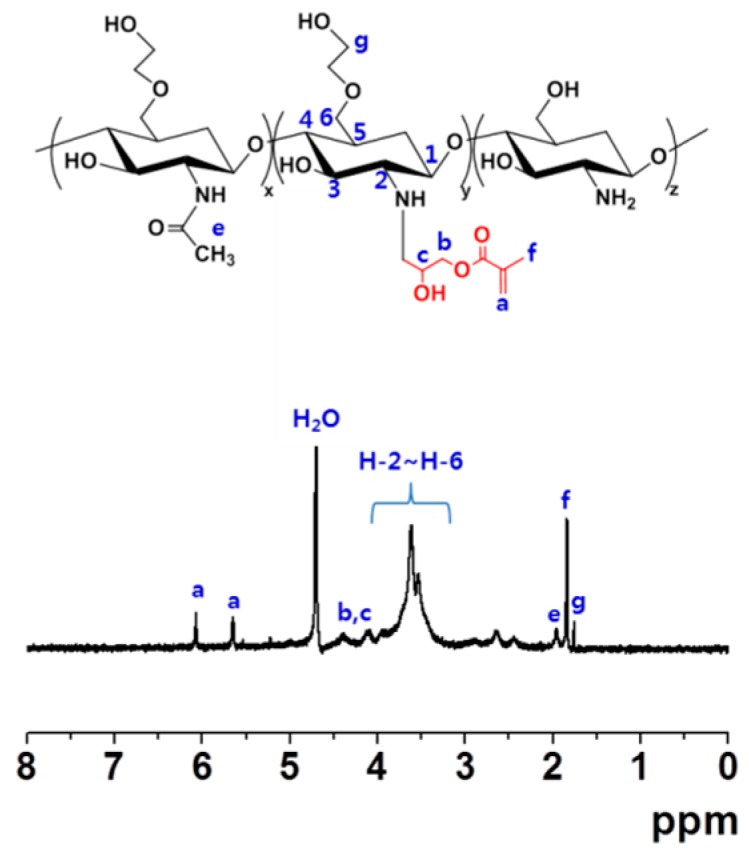
^1^H-NMR spectra of glycidyl methacrylate (GM)-conjugated glycol chitosan (GC) measured in D_2_O.

Conjugation of glycidyl methacrylate (GM) to GC was confirmed by ^1^H-NMR measurement (Figure 1). Several peaks at 1.83, 4.11, 4.39, 5.65 and 6.08 ppm were assigned for GM. Multiple peaks at 1.75, 1.95 and 3.24–3.96 ppm were observed for GC. The results demonstrated that GM was successfully conjugated to GC [8].

### 2.2. Characterization of GC Hydrogels Formed by Different Concentrations of GC

**Figure 2:**
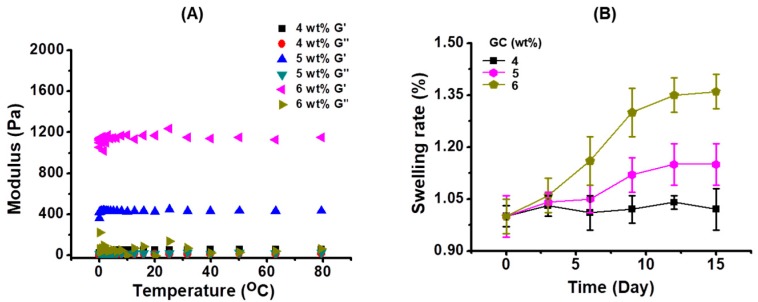
(**A**) Storage/loss (G′/G′′) moduli and (**B**) swelling ratio of GC hydrogels prepared by 4, 5, and 6 wt % of GC. The hydrogels showed larger storage modulus and loss. In addition, their swelling ratio increased with an increase of GC concentration.

Figure 2A shows the storage (G’) and loss (G”) moduli of GC hydrogels formed by different concentrations of GC (4, 5 and 6 wt %). The storage modulus was larger than the loss modulus, indicating hydrogel formation. The storage modulus increased as the concentration increased. This may be attributed to the concentration and the three-dimensional network formation [14]. The swelling ratio of GC hydrogels on different concentrations is shown in Figure 2B. The swelling ratio increased as the concentration increased, demonstrating that the large amount of cross-linked GC polymers at a specific photo-initiator concentration may be swelled by invading water molecules in the network [14].

### 2.3. SEM Images and Degradation Ratio of Hydrogel Prepared by 4 wt % GC as a Function of Time

**Figure 3:**
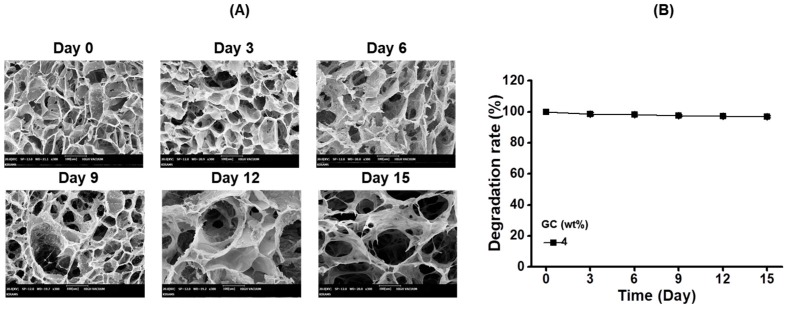
(**A**) SEM images (×300) and (**B**) degradation rate of GC hydrogel (4 wt %) examined at 0, 3, 6, 9, 12 and 15 days. The hydrogels showed a porous, interconnected structure without mass loss as a function of time.

Figure 3 shows the SEM images and degradation rates of GC hydrogel (4 wt %) examined at 0, 3, 6, 9, 12 and 15 days. The morphologies exhibited porous, interconnected structures regardless of time flow. Although the pore sizes at day 12 and 15 seemed larger than the other days, the difference in the sizes was actually marginal. In addition, the degradation rates remained unchanged.

### 2.4. Cytotoxicity of CUR on L-929 Cells

**Figure 4:**
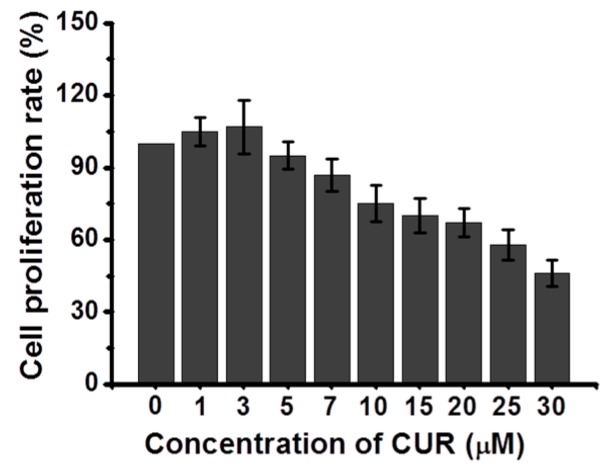
Cytotoxicity of curcumin (CUR) as a function of concentration examined by cell counting kit (CCK)-agent. 5 μM of concentration was a threshold point.

Figure 4 shows the cytotoxicity of CUR on L-929 cells as a function of CUR concentration. The cytotoxicity remained unchanged for up to 5 μM and decreased thereafter. This indicated that a CUR concentration below 5 μM is cytocompatible [15,16].

### 2.5. Examination of the Inclusion Complex between β-CD and CUR

**Figure 5:**
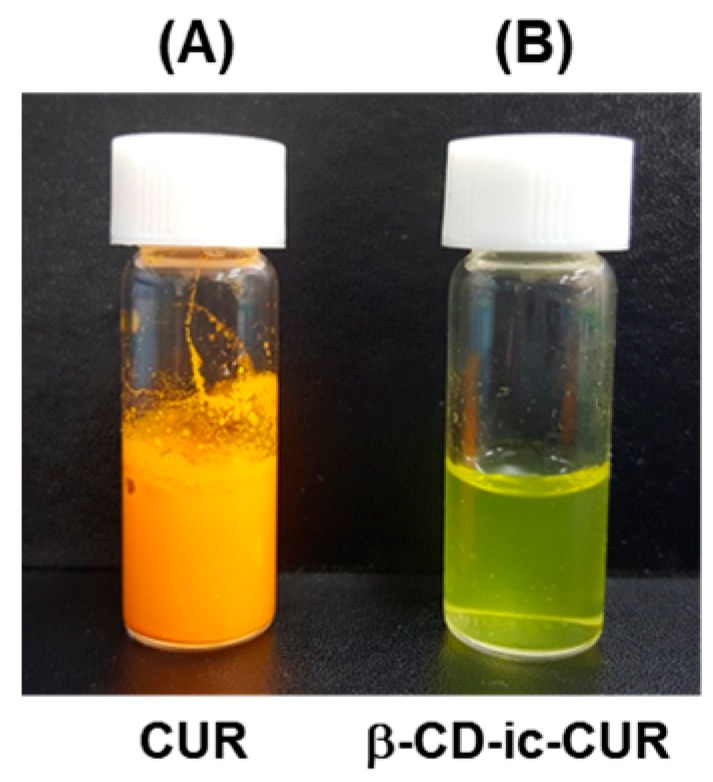
Photographs showing (**A**) CUR and (**B**) inclusion complex formation between β-CD and CUR in water.

The inclusion complex was confirmed by simply mixing the aqueous solutions of β-CD and CUR (Figure 5). As shown in Figure 5A, CUR in water was not dissolved due to its poor water-solubility, but dispersed. On the other hand, CUR dissolved well in water, because β-CD could form an inclusion complex (Figure 5B) [17].

### 2.6. Surface and Cross-Sectional Morphologies

**Figure 6:**
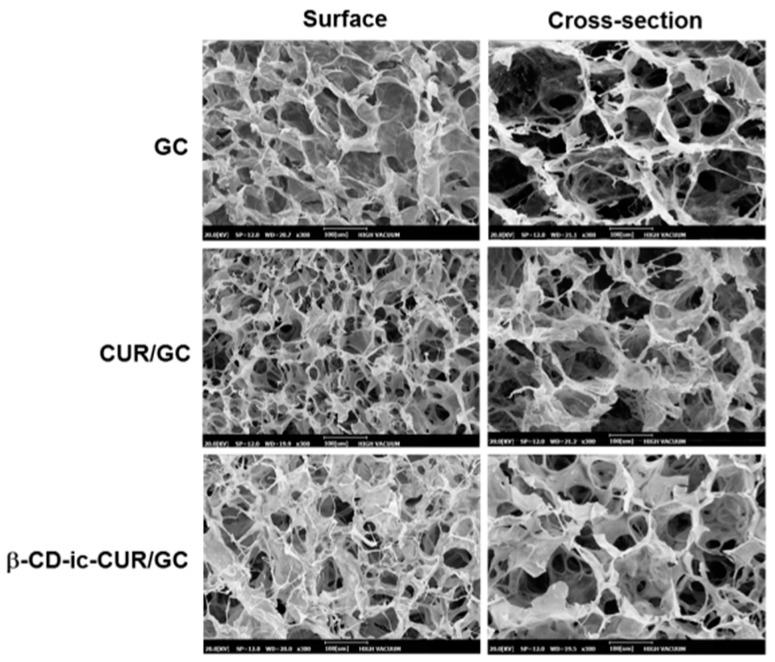
SEM images of freeze-dried GC, CUR/GC and β-CD-ic-CUR/GC hydrogels observed at ×300. A porous structure was observed in the surface and cross-sectional morphologies of the hydrogel samples. GC—glycol chitosan hydrogel; CUR/GC—curcumin loaded glycol chitosan hydrogel; β-CD-ic-CUR/GC—curcumin complexed β-CD loaded glycol chitosan hydrogel.

The surface and cross-sectional morphologies of freeze-dried GC, CUR/GC, and β-CD-ic-CUR/GC hydrogels were observed by SEM (Figure 6). A porous, interconnected structure was observed on the surface and in the cross-section. There was a marginal difference in the size of pores on the surface and in the cross-section. Furthermore, CUR and β-CD-ic-CUR were not observed in CUR/GC and β-CD-ic-CUR/GC, respectively. These observations could be attributed to the small sizes of CUR and β-CD-ic-CUR.

### 2.7. Storage and Loss Moduli

**Figure 7:**
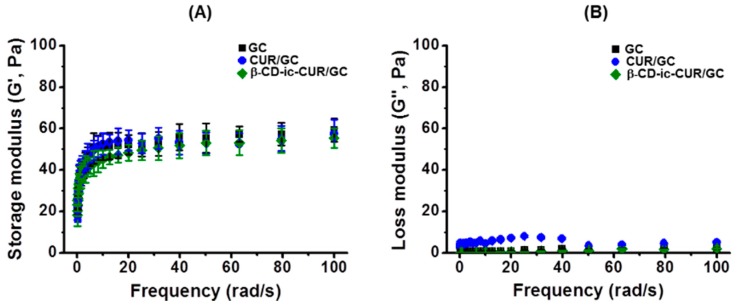
(**A**) Storage (G′) and (**B**) loss (G′′) moduli of the GC, CUR/GC and β-CD-ic-CUR/GC hydrogels measured as a function ranging from 0 rad/s to 100 rad/s, by rheometry. The storage moduli of the hydrogel samples were greater than their loss moduli, indicating hydrogel formation. GC, glycol chitosan hydrogel; CUR/GC, curcumin-loaded glycol chitosan hydrogel; β-CD-ic-CUR/GC, curcumin-conjugated β-CD-loaded glycol chitosan hydrogel.

The storage (G′) and loss (G′′) moduli of the samples were evaluated as a function of frequency from 0 rad/s to 100 rad/s, by rheometry (Figure 7). After visible-light irradiation, the storage moduli of the samples were greater than their loss moduli. This indicated that the samples were converted from sol state to gel state [8]. The storage and loss moduli of the samples were between 40 Pa and 60 Pa, and below 10 Pa, respectively, and no significant differences between the samples were observed. At 100 rad/s, the storage moduli of GC, CUR/GC, and β-CD-ic-CUR/GC were 59.2 Pa, 57.5 Pa, and 55.5 Pa, respectively.

### 2.8. Fourier-Transform Infrared Spectroscopic (FT-IR) Analysis

**Figure 8:**
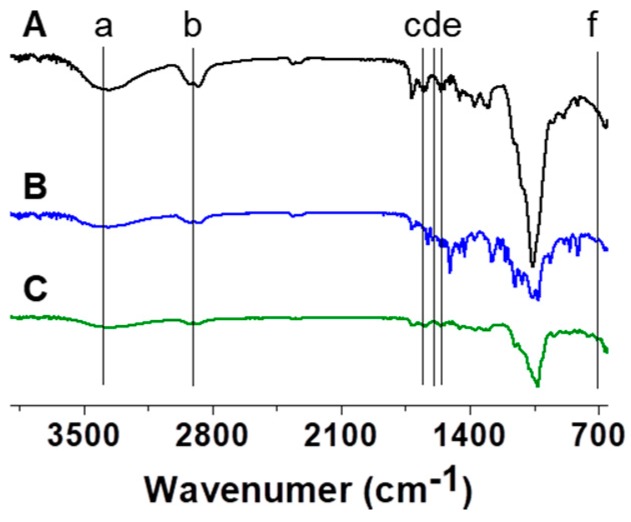
FTIR spectra of the lyophilized (**A**) GC, (**B**) CUR/GC, and (**C**) β-CD-ic-CUR/GC hydrogels obtained from 650 cm^−1^ to 3900 cm^−1^. OH stretching overlapped with NH stretching at 3200~3400 cm^−1^ (a), CH stretching at 2800~3000 cm^−1^ (b), C=O stretching of amide I at 1653 cm^−1^ (c), NH bending of amine and amide II at 1558 cm^−1^, (e) and cis CH vibration of the aromatic ring and symmetric aromatic-ring stretching vibration of CUR (d and f).

Figure 8 shows the FT-IR spectra of the GC, CUR/GC, and β-CD-ic-CUR/GC hydrogels obtained from 650 cm^−1^ to 3900 cm^−1^. GC exhibited certain unique absorption peaks, including the overlapping of OH stretching with NH stretching at 3200~3400 cm^−1^, CH stretching at 2800~3000 cm^−1^, C=O stretching of amide I at 1653 cm^−1^, and NH bending of amine and amide II at 1558 cm^−1^ [18]. The two peaks at 713 cm^−1^ and 1602 cm^−1^ of CUR/GC and β-CD-ic-CUR/GC were attributed to the cis CH vibration of the aromatic ring and the symmetric aromatic-ring stretching vibration of CUR, respectively [19]. In particular, the differences among the absorption peaks were attributed to the β-CD-CUR inclusion complex formation [19].

### 2.9. Thermal Gravimetric Analysis (TGA)

**Figure 9:**
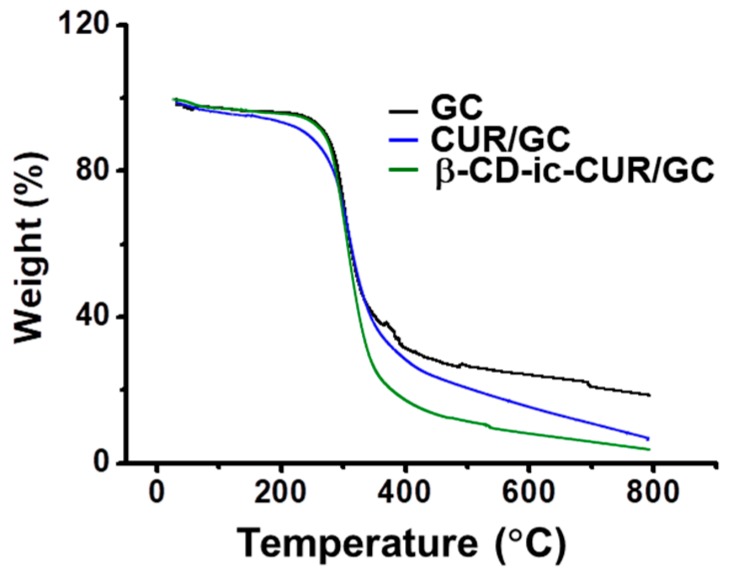
TGA curves of the lyophilized GC, CUR/GC, and β-CD-ic-CUR/GC hydrogels analyzed from 10 °C to 800 °C. The hydrogels exhibited no remarkable thermal behavior owing to the modest quantity of CUR or β-CD-ic-CUR.

The thermal behavior of GC, CUR/GC, and β-CD-ic-CUR/GC were analyzed by TGA from 25 °C to 800 °C in a N_2_ atmosphere (Figure 9). The samples exhibited weight loss in two curves below 100 °C and at 280 °C. The first and second signals indicated absorbed-water evaporation and chitosan decomposition, respectively [20]. Despite the addition of a specific quantity of CUR or β-CD-ic-CUR, their unique thermal behavior was not observed. This could be attributed to their modest quantities.

### 2.10. Release Behavior of CUR

**Figure 10:**
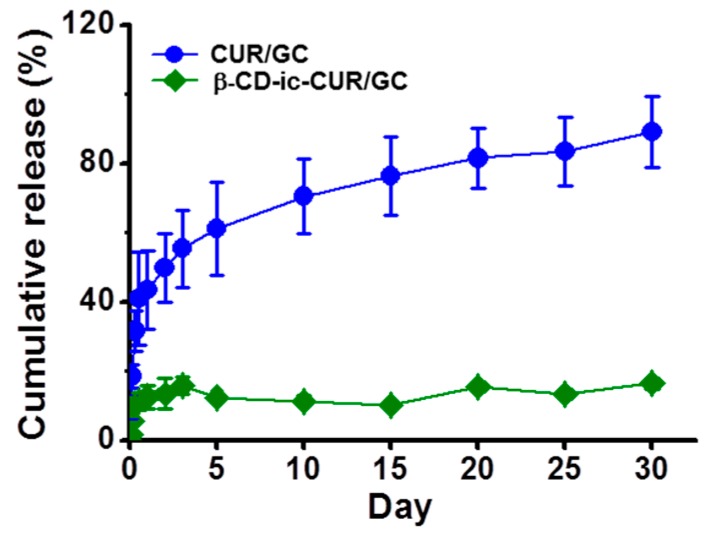
Release behavior of CUR from the CUR/GC and β-CD-ic-CUR/GC hydrogels analyzed in PBS (pH 7.4) for 30 days by UV-Vis spectroscopy. β-CD-ic-CUR/GC exhibited controlled CUR release behavior along with an initial burst within 12 h. Contrarily, controlled release was not observed in the CUR/GC hydrogel. GC, glycol chitosan hydrogel; CUR/GC, curcumin-loaded glycol chitosan hydrogel; β-CD-ic-CUR/GC, curcumin-conjugated β-CD-loaded glycol chitosan hydrogel.

The cumulative percentages of CUR release from CUR/GC and β-CD-ic-CUR/GC were evaluated for a release period of 30 days in phosphate buffered saline (PBS, pH 7.4), as shown in Figure 10. β-CD-ic-CUR/GC exhibited controlled CUR release in a sustained manner, and the release became 90% after 30 days, after an initial burst within 12 h. However, the cumulative percentage of CUR release from CUR/GC was below 17% throughout the release period. This indicated that β-CD served as an excipient for controlling CUR release [21].

### 2.11. Proliferation Rate of L-929 Cells

**Figure 11:**
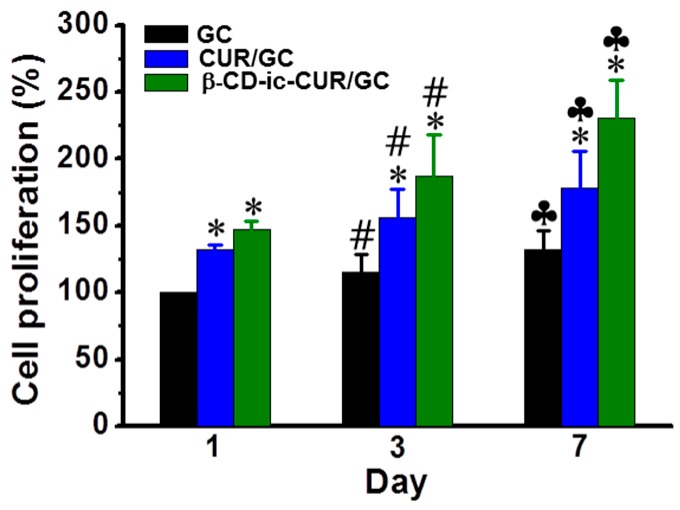
Proliferation of the L-929 cells cultured on the GC, CUR/GC, and β-CD-ic-CUR/GC hydrogels, evaluated by the CCK-8 assay. * *p* < 0.05, significance of CUR/GC and β-CD-ic-CUR/GC compared with GC; ^#^ *p* < 0.05, significance of day 3 compared with day 1; ^♣^ *p* < 0.05, significance of day 7 compared with day 1. The cells proliferated actively on the β-CD-ic-CUR/GC hydrogel throughout the culture periods, compared with GC and CUR/GC. GC, glycol chitosan hydrogel; CUR/GC, curcumin-loaded glycol chitosan hydrogel; β-CD-ic-CUR/GC, curcumin-conjugated β-CD-loaded glycol chitosan hydrogel.

The cell proliferation rates of L-929 cells cultured on GC, CUR/GC, and β-CD-ic-CUR/GC were examined using a cell counting ket (CCK) assay (Figure 11). CUR-loaded samples showed higher cell proliferation rate than did GC-loaded samples. Moreover, cells cultured on β-CD-ic-CUR/GC proliferated more actively than those on CUR/GC, which can be attributed to the water-solubility of CUR.

### 2.12. In Vivo Wound-Healing Analysis

**Figure 12:**
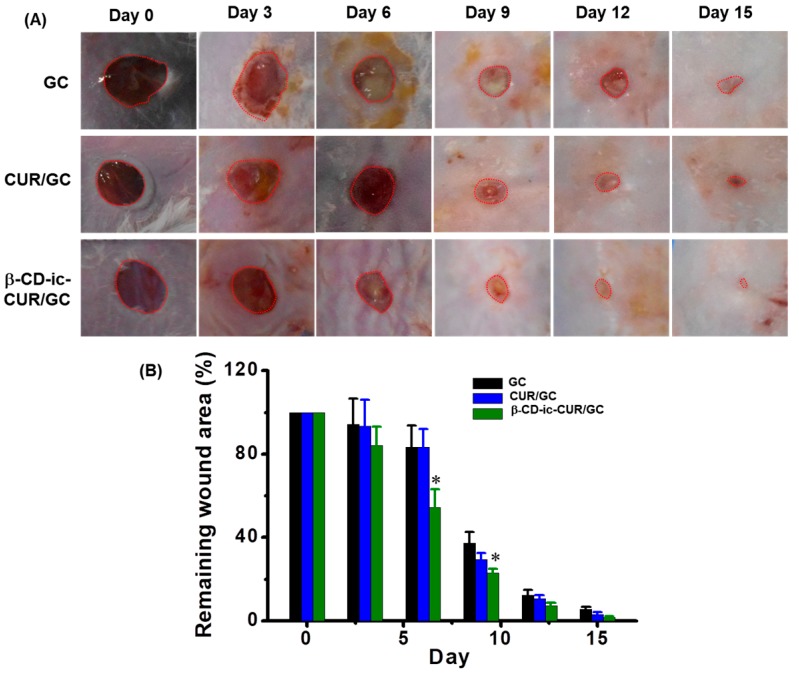
(**A**) Photographs and (**B**) remaining area (%) of the wounds after 0, 3, 6, 9, 12, and 15 days of hydrogel treatment. * *p* < 0.05. The most rapid wound healing was observed in the β-CD-ic-CUR/GC-treated wound throughout the healing periods. GC, glycol chitosan hydrogel; CUR/GC, curcumin-loaded glycol chitosan hydrogel; β-CD-ic-CUR/GC, curcumin-conjugated β-CD-loaded glycol chitosan hydrogel.

The macroscopic appearance and the remaining wounded areas treated with GC, CUR/GC, β-CD-ic-CUR/GC, and control were examined once every three days for 15 days (Figure 12). Impairment in the macroscopic appearance of the wounds treated with the GC-based samples was observed, compared with the control. Moreover, the CUR-loaded samples showed better wound-healing effects than GC. β-CD-ic-CUR/GC, in particular, exhibited rapid accelerating effects on wound-healing. To further investigate the wound-healing effect, the remaining wounded area was calculated using an Image J program (National Institutes of Health, Bethesda, MD, USA). Treatment with the GC-based hydrogels resulted in more rapid wound healing than that with the control throughout the healing period. β-CD-ic-CUR/GC, in particular, induced the most rapid acceleration of wound healing among the GC-based hydrogels.

### 2.13. Histological Analyses

**Figure 13:**
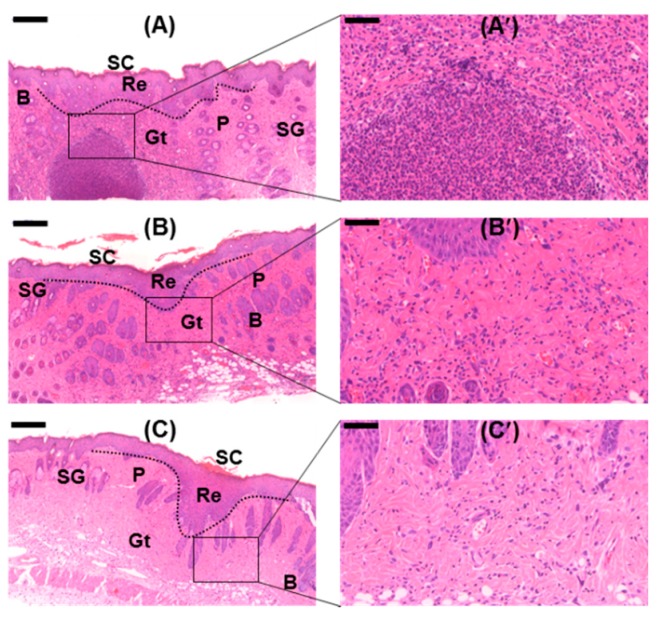
H&E-stained images of the wounds after 15 days of hydrogel treatment; (**A**,**A′**) GC-treated wound, (**B**,**B′**) CUR/GC-treated wound, and (**C**,**C′**) β-CD-ic-CUR/GC-treated wound. Black scale bar is 200 μm. The lowest number of inflammatory cells was observed in the β-CD-ic-CUR/GC-treated wound. GC, glycol chitosan hydrogel; CUR/GC, curcumin-loaded glycol chitosan hydrogel; β-CD-ic-CUR/GC, curcumin-conjugated β-CD-loaded glycol chitosan hydrogel.

**Figure 14:**
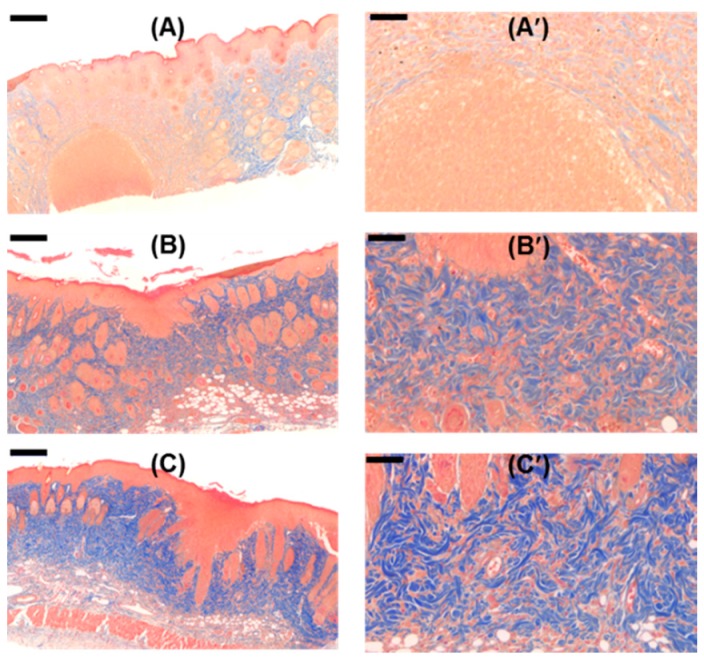
Masson’s trichrome-stained images of the wounds after 15 days of hydrogel treatment; (**A**,**A′**) GC-treated wound, (**B**,**B′**) CUR/GC-treated wound, and (**C**,**C′**) β-CD-ic-CUR/GC-treated wound. Black scale bar is 200 μm. The highest extent of collagen synthesis was observed in the β-CD-ic-CUR/GC-treated wound. GC, glycol chitosan hydrogel; CUR/GC, curcumin-loaded glycol chitosan hydrogel; β-CD-ic-CUR/GC, curcumin-conjugated β-CD-loaded glycol chitosan hydrogel.

The histological analyses of the wounds treated with the GC-based hydrogels for 15 days, by H&E and Masson’s trichrome (MT) staining were conducted, and compared with the control (Figure 13 and Figure 14). The wound samples commonly exhibited scabs (SC), re-epithelialization (Re), granulation tissues (Gt), sebaceous glands (SG), papillae (P), and bulbs (B). The magnified H&E-stained images showed that the GC-based hydrogel-treated samples exhibited a lower number of inflammatory cells than the control-treated samples [8]. Remarkable collagen synthesis was observed in the GC-based hydrogel-treated samples. Treatment with β-CD-ic-CUR/GC, in particular, increased the extent of collagen synthesis. The results indicated that improved CUR water solubility accelerated the healing of the soft-tissue defects.

## 3. Discussion

Wound healing is a systematic process, which occurs sequentially, beginning from injury through inflammation, proliferation, and remodeling [22]. The primary purpose of wound healing is the repair of defective soft tissue with minimal scarring. Many wound-dressing systems have been developed for achieving this [2]; however, challenges to the healing of, specifically, soft-tissue defects in open fractures, remain. Hydrogels might be attractive for use in ideal dressings for surgical or highly damaged orthopedic injuries, because they can contain drugs and growth factors that help in accelerating wound healing [11]. Additionally, hydrogel systems allow for the protection of wounds from surgical site infections by maintaining moist environments around the wounds [4]. The moisture induces the initiation of healing phases, such as granulation, epidermal repair, and removal of dead tissue, resulting in the promotion of wound healing [22].

Chitosan hydrogels are potentially beneficial materials for wound healing. Chitosan can form hydrogel systems with three-dimensional networks by conjugating its amine functional groups with cross-linkers. Furthermore, chitosan can regulate macrophage M1/M2 polarization [10]. Macrophages play important roles in wound healing by performing many functions, including host defense, promotion and resolution of inflammation, removal of apoptotic cells, and promotion of cell proliferation and tissue regeneration [23]. Nevertheless, chitosan has limitations in clinical application, because of its poor solubility [24]. GC, which is a chitosan derivative, is water-soluble, because the intramolecular hydrogen bonds formed among the C_3_-OH, C_6_-OH, and NH_2_ groups are broken by glycol conjugation at the OH groups [25]. Therefore, GC was employed in this study.

In this study, we prepared photocured GC-based hydrogels with controlled CUR release using a visible light-curing system, and evaluated their efficacy in the acceleration of wound healing in vivo. GM is a possible functional group for photocuring, because it can conjugate with the hydroxyl group of GC through the ring-opening reaction of the epoxy group [8]. As expected, the reaction was conducted successfully, as confirmed by ^1^H-NMR analysis (Figure 1).

The analysis of the storage moduli and swelling ratios of the GC hydrogels is an essential process in the application of tissue-engineered scaffolds. The characterization is known to be affected by the concentration of the polymer and its crosslinking density [14], as shown in Figure 2. Collagen-based hydrogels with storage moduli of approximately 78 Pa have been used extensively for the acceleration of wound healing [26], which has inspired researchers to develop efficacious wound dressings with appropriate mechanical properties. Hydrogels with appropriate mechanical properties should provide platforms for preventing secondary infection, and facilitating the adhesion, proliferation, and differentiation of cells. In this study, we selected 4 wt %-GC hydrogel for in vitro and in vivo analyses, because the storage modulus of the hydrogel might be appropriate for the acceleration of wound healing.

At low concentrations (between 1 and 5 μM), CUR stimulates the migration and proliferation of cells, while at higher concentrations, it inhibits its effects [15]. Moreover, at high concentrations (>25 μM), CUR induces the apoptosis of fibroblasts [16], which was consistent with our results (Figure 3). Hence, a specific CUR concentration (3 μM) was used in this study.

In the inflammatory stage, CUR inhibits the activity of the transcription factor, NF-κB, and reduces the production of the cytokines, TNF-a and IL-1, thereby reducing inflammation [27]. In the proliferation stage, CUR enhances fibroblast migration, granulation tissue formation, collagen deposition, and re-epithelialization [28]. In the remodeling stage, CUR increases wound contraction by increasing TGF-β production and fibroblast proliferation. Despite these functions, CUR has poor water solubility, resulting in low bioavailability. β-CD can improve its water solubility by forming an inclusion complex with CUR, which has a binding constant of 134.002 M^−1^ [17]. Therefore, β-CD was used for increasing the water solubility of CUR, and the complex was loaded in a GC hydrogel. The wound-healing effects of the β-CD-ic-CUR/GC hydrogel were compared with those of GC and CUR/GC.

The controlled release of drugs or growth factors in a sustained manner is a prerequisite for the promotion of wound healing. Some research groups have reported that systems for the controlled release of drugs or growth factors are beneficial for managing chronic wounds together with the direct application of high antibiotic doses combined with plastic dressings on orthopedic injuries, as it is not necessary to treat them repeatedly, thereby providing comfort to patients [29,30,31]. Hydrogel systems with porous structures were observed to control the release of biomolecules owing to their biodegradability, swelling, and diffusion behavior [32,33,34,35,36]. The GC-based hydrogels exhibited interconnected, porous structures (Figure 4). Furthermore, the degradation rate of a polymer hydrogel affects the release behavior, and therefore the determination of the degradation rate can be a requirement in the application of tissue-engineered scaffolds. The degradation rate of the GC hydrogel remained unchanged during the 15 days of incubation, indicating that CUR was released because of the diffusion behavior (Figure 2, Figure 3, Figure 4, Figure 5, Figure 6, Figure 7, Figure 8, Figure 9 and Figure 10).

Apart from the porous structure, β-CD, as an excipient, might be an important factor for controlled drug release. Excipients used for increasing the water solubility of hydrophobic drugs can induce improved drug release in a sustained manner [21]. As expected, the porous chitosan hydrogels synthesized using the visible light-curing system, along with β-CD as an excipient, facilitated controlled CUR release in a sustained manner for 30 days (Figure 10).

Fibroblasts affect the normal wound healing process by inducing the degradation of fibrin clots and the formation of the new extracellular matrix (ECM) and collagen, to support myofibroblasts with effective wound healing [37]. Hydrogel systems would provide appropriate frameworks that can increase the adherence to and proliferation of fibroblasts. Additionally, CUR induces rapid wound healing by recruiting fibroblasts for migration and proliferation. The CUR-loaded samples showed higher cell-proliferation rates than the GC-loaded samples (Figure 11).

Owing to the merits of mouse models, such as availability, low cost, and small size, Bagg Alvino (inbred research mouse stratin) (BALB/c) mice were used in this study. We developed a mouse model by inflicting a circular 5-mm wide wound using a punch, and wound healing was observed for 15 days. β-CD-ic-CUR/GC exhibited better wound-healing effects than the control, GC, and CUR/GC (Figure 12). This finding could be attributed to the improved water solubility of CUR and modulation of macrophages by GC.

H&E and Masson’s trichrome staining are histopathological techniques for analyzing cellular behavior and collagen synthesis during wound healing, respectively [38,39]. H&E staining is the most commonly used technique in histopathological studies; however, it cannot be used for distinguishing important histopathological changes during wound healing, such as collagen synthesis [38,39]. Masson’s trichrome staining was adopted to analyze collagen synthesized during wound healing [38]. The GC-based hydrogel treated wounds commonly exhibited re-epithelialization, angiogenesis, and collagen synthesis (Figure 13 and Figure 14). During wound healing, the promotion of re-epithelialization, angiogenesis, and collagen synthesis plays an important role in the acceleration of the healing [23]. This indicated that the wounds were healed continuously. Among the GC-based hydrogels, treatment with GC resulted in the largest number of inflammatory cells. However, treatment with CUR/GC- and β-CD-ic-CUR/GC decreased the number of inflammatory cells, along with increased collagen synthesis. The highest extent of collagen synthesis was observed in the β-CD-ic-CUR/GC-treated wound, in particular.

Wound healing is mainly classified into three phases, namely, inflammation, proliferation, and remodeling [23]. In the inflammatory phase, the wound is covered with a blood clot, and neutrophils invade the wound [23]. In the proliferation phase, the neutrophils undergo apoptosis, and the wound is filled with macrophages [23]. Moreover, endothelial cells and fibroblasts migrate into the clot and the wound site, respectively, for the formation of new blood vessels [23]. Finally, wound healing occurs by filling with granulation tissue, transforming fibroblasts into myofibroblasts for wound contraction, and collagen synthesis [23]. These facts indicated that the GC- and CUR-loaded hydrogel-treated wounds were healed in the inflammatory and remodeling phases, respectively. Moreover, β-CD-ic-CUR/GC exhibited a more rapid wound-healing effect than CUR/GC. These results provided evidence supporting the proposition that an increase in the water solubility of CUR contributes to the acceleration of wound healing.

In this study, we confirmed the feasibility of GC-based hydrogels with improved water solubility of CUR in the acceleration of wound healing, and hence, the wound healing-effect will be investigated by controlling the solubility and rate of CUR release from GC hydrogels, in a future study. Additionally, there have been a few studies on the different forms of glycol chitosan, such as films, sponges, and matrices, for applications in wound healing, to date. Therefore, future studies will also focus on the development of the optimum formulation of GC-based scaffolds for clinical wound-healing applications.

## 4. Materials and Methods

### 4.1. Materials

Glycol chitosan (GC, ≥60% (by titration), crystalline, Mw ≅ 585,000 g/mol), glycidyl methacrylate (GM), and curcumin (CUR) were purchased from Sigma-Aldrich (St. Louis, MO, USA). 4-(4,6-Dimethoxy-1,3,5-triazin-2-yl)-4-methylmorpholinium chloride (DMT-MM) was obtained from Wako Pure Chemical Industries, Ltd. (Osaka, Japan). Dialysis tubing (Spectrum Laboratories Inc., Rancho Dominguez, CA, USA) was used for purification. The mouse L-929 fibroblast cell line was supplied by Korean Cell Line Bank (Seoul, Korea). All the chemicals were used as received without further purification.

### 4.2. Preparation of GM-Conjugated GC (GM-GC)

GM-GC was prepared as reported previously [8]. The GC solution (0.003 mmol, 1.5 g) was acidic, and its pH was adjusted to 9 with 1 N HCl. Then, GM (0.05 mmol, 7 mg) was added, and the reaction was carried out at room temperature for two days, and then neutralized. Then the solution was dialyzed (cut off: 20 kDa), and lyophilized at −90 °C for seven days. The solid thus formed was stored at −20 °C until use. The final product was characterized by ^1^H-NMR analysis (Bruker Avance 400; Bruker Corp., Manning Park, Billerica, MA, USA) with D_2_O.

### 4.3. Preparation and Characterization of Visible Light-Cured GC Hydrogel (GC) as a Function of Concentration

**Figure 15:**
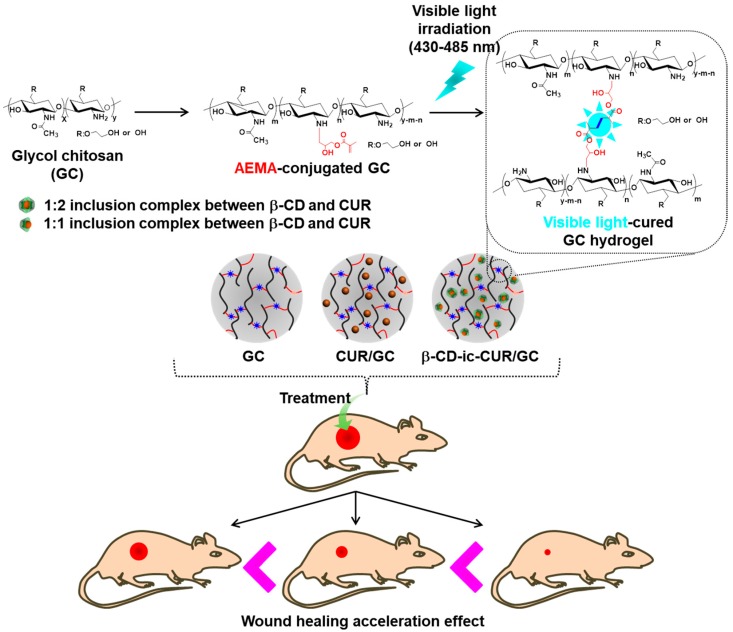
Schematic illustration of GC, CUR/GC, and β-CD-ic-CUR/GC hydrogels, and their applications in skin wound healing in a mouse model.

GC hydrogel was prepared using three concentrations of GC with a visible light-curing system, as shown in Figure 15. Riboflavin (12 μM) was added to each GM-GC solution (4%, 5% and 6% *w*/*v*). After homogeneous dispersion, the mixture was irradiated with visible light (430~485 nm, 2100 mW/cm^2^, light-emitting diode (LED) curing light, Foshan Keyuan Medical Equipment Co., Ltd., Guangdong, China) for 200 s. The swelling ratio of the hydrogels was measured in PBS (pH 7.4) at the predetermined time intervals (0, 3, 6, 9, 12 and 15 days). At each time interval, the hydrogels were extracted and washed with distilled water three times. The water on the surface of the hydrogels was removed and the hydrogels were weighed. The swelling ratio was calculated by the ratio of the swollen weight to the initial weight of the hydrogels. The degradation rate of hydrogel prepared using 4 wt % GC was also examined at the predetermined time intervals. At each time interval, the hydrogel was extracted and washed with distilled water three times. The rinsed hydrogel was then lyophilized, and afterward, the hydrogel was weighed. The degradation ratio was expressed as the ratio of the remaining weight to the initial weight of the hydrogel.

### 4.4. Formation of Inclusion Complex between β-CD and CUR (β-CD-ic-CUR)

β-CD (1 mmol, 1.13 g) and CUR (1 mmol, 368 mg) were dissolved in distilled water (20 mL) and acetone (5 mL), respectively. The CUR solution was poured into the aqueous β-CD solution with continuous agitation, and stirred until the acetone evaporated completely. After filtration, the β-CD-ic-CUR was lyophilized at −90 °C, and stored in a desiccator set at one atmosphere until use.

### 4.5. Analysis of CUR Cytotoxicity

The L-929 cells (1 × 10^4^ cells/well) in 96-well plates were treated with CUR at various concentrations (1, 3, 5, 7, 10, 15, 20, 25, and 30 μM), and cultured with Eagle’s minimal essential medium, supplemented with 5% fetal bovine serum (FBS), penicillin (100 units/mL), and streptomycin (100 μg/mL) for 1, 3, and 7 days. The cells were treated with the CCK-8 reagent (100 μL), and incubated for 2 h. The supernatant was collected from each well, and the optical density was measured using a microplate reader (SpectraMax^®^ i3; Molecular Devices, Sunnyvale, CA, USA) at 450 nm.

### 4.6. Preparation of CUR and β-CD-ic-CUR-Loaded GC Hydrogels (CUR/GC and β-CD-ic-CUR/GC)

Prior to irradiation with visible light, CUR (3 μM) or β-CD-ic-CUR (3 μM for CUR) was loaded in each GM-GC solution (Figure 8). The subsequent procedures were conducted by following the same methods as described in Section 4.3.

### 4.7. Scanning Electron Microscopy (SEM)

Samples on metal mounts were gold-coated using an ion sputter coater (COXEM Co. Ltd., Daejeon, Korea). The surface and cross-sectional morphology of the samples was examined by SEM (CX-200TM; COXEM Co. Ltd., Daejeon, Korea) at 20.0 kV, and observed at 300× and 500× magnification.

### 4.8. Rheological Analysis

The rheological properties of the samples, as functions of frequency, were analyzed using an AR 2000 EX rheometer (TA instruments, New Castle, DE, USA) set with a cone and plate geometry of a diameter of 4 cm and cone angle of 1°. The hydrogels (370 μL) were subjected to analysis by a rotational cone and plate rheometer at controlled shear rates. The storage (G′) and loss (G′′) moduli were measured at 37 °C at a range of 0 to 100 Hz.

### 4.9. FTIR Analysis

An FTIR spectrometer (Nicolet iS 10, Thermo Fisher Scientific, Waltham, MA, USA) was employed to confirm the structural analysis of GC, CUR/GC, and β-CD-ic-CUR. The measurement was conducted at 0.5 cm^−1^ resolution ranging from 700 cm^−1^ to 3900 cm^−1^.

### 4.10. TGA Measurement

TGA (Q600, TA Instruments, New Castle, DE, USA) was employed to confirm the thermal behavior of GC, CUR/GC, and β-CD-ic-CUR. The measurement was conducted under a balance sensitivity of 0.1 μg, 0.1 °C to 25/min, and a sensitivity of 0.001 °C, ranging from 10 °C to 800 °C.

### 4.11. Analysis of CUR Release

The CUR/GC or β-CD-ic-CUR/GC (adjusted to 3 μM CUR) hydrogel was transferred onto a Corning 24-well Transwell polyester membrane. After placing in 24-well plates, the samples were incubated with 2 mL of PBS (pH 7.4) in an incubator set at 37 °C. At predetermined time intervals, a volume of PBS was replaced with fresh PBS. The withdrawn PBS was analyzed by ultraviolet-visible (UV-Vis) spectroscopy at 425 nm.

### 4.12. In Vitro Cell Proliferation

Hydrogel precursor solutions (150 μL) were added to 96-well plates, and photocured with visible-light irradiation. After seeding the L-929 cells (1 × 10^4^ cells/well) onto the hydrogel surfaces, Eagle’s minimal essential medium, supplemented with 5% FBS, penicillin (100 units/mL), and streptomycin (100 μg/mL), was added to each well, and incubated for 1, 3, and 7 days. At each time point, the cells were treated with the CCK-8 reagent (100 μL), and incubated for 2 h. The supernatant was collected from each well, and the optical density was measured using a microplate reader (SpectraMax^®^ i3; Molecular Devices, Sunnyvale, CA, USA) at 450 nm.

### 4.13. In Vivo Wound-Healing Study

All animal experimental protocols were approved by the Institutional Animal Care and Use Committee of the Chonbuk National University Laboratory Animal Center (CUH 2017-15). Twelve BALB/c mice (20 g, *n* = 6) were used for the in vivo study of the healing of full-thickness skin wounds. All the animals were anesthetized with Ketara^®^ (Yuhan Yanghang, Seoul, Korea) (50 mg/kg) and Rompun^®^ (Bayer Korea, Seoul, Korea) (5 mg/kg), and their backs were shaved. A 5-mm-wide biopsy punch was used for inflicting a wound on the dorsal side of the skin. Two wounds were inflicted on each rat. Twelve mice were used for each time point. GC, CUR/GC (corresponding to 3 μM CUR), and β-CD-ic-CUR/GC (corresponding to 3 μM CUR) (200 μL each) hydrogels were applied randomly on the wounded area (Figure 15). Changes in the wounded region were evaluated using an ImageJ program (Version k 1.45, National Institutes of Health, Bethesda, MD, USA) on days 0, 3, 6, 9, 12, and 15 after initial wound infliction. On day 15, the hydrogel sample-treated animals were euthanized by exposure to 100% CO_2_. The wounded region and surrounding skin and muscle tissues were removed, and fixed in 10% neutral buffered formalin. The tissue samples were embedded in paraffin, and sectioned for analysis by hematoxylin and eosin (H&E) and Masson’s trichrome (MT) staining.

### 4.14. Histological Analysis

The excised wounded tissues were fixed in 10% neutral formaldehyde. The fixed tissues were embedded in paraffin, and sectioned at 3 μm. The sections were analyzed histologically by H&E and MT staining. The stained slides were observed by fluorescence microscopy (IX71 inverted microscope, Olympus, Tokyo, Japan).

### 4.15. Statistical Analysis

All quantitative data were expressed as means ± standard deviation. One-way analysis of variance (ANOVA) was conducted using SPSS (SPSS Inc., Chicago, IL, USA). A *p* value < 0.05 was considered statistically significant. The number of each sample for the in vivo animal study was calculated using MedCalc (MedCalc Software bvba, Ostend, Belgium) using α (*p* = 0.05) and power (1 − β = 0.8).

## 5. Conclusions

In this study, we synthesized visible light-cured GC hydrogels, containing the inclusion complex of β-CD and CUR, and their efficacy in accelerating wound healing was compared with that of CUR/GC. Wound healing was accelerated by increasing the solubility of CUR in water together with its controlled release in a sustained manner. The results suggested that β-CD-ic-CUR/GC might have potential for clinical use in wet wound dressings for soft-tissue defects.
